# A Miniature Integrated Multimodal Sensor for Measuring pH, EC and Temperature for Precision Agriculture

**DOI:** 10.3390/s120608338

**Published:** 2012-06-15

**Authors:** Masato Futagawa, Taichi Iwasaki, Hiroaki Murata, Makoto Ishida, Kazuaki Sawada

**Affiliations:** 1 Head Office for “Tailor-Made and Baton-Zone” Graduate Course, Toyohashi University of Technology, 1-1, Hibarigaoka, Tempaku-cho, Toyohashi, Aichi 441-8580, Japan; 2 Department of Electrical and Electronic Information Engineering, Toyohashi University of Technology, Aichi 441-8580, Japan; E-Mails: iwasaki-t@int.ee.tut.ac.jp (T.I.); murata-h@int.ee.tut.ac.jp (H.M.); ishida@ee.tut.ac.jp (M.I.); sawada@ee.tut.ac.jp (K.S.); 3 Electronics-Inspired Interdisciplinary Research Institute (EIIRIS), Toyohashi University of Technology, Aichi 441-8580, Japan; 4 Core Research for Evolutional Science and Technology, Japan Science and Technology Agency, 7, Gobancho, Chiyoda-ku, Tokyo 102-0076, Japan

**Keywords:** multimodal sensor, electrical conductivity sensor, pH sensor, temperature sensor, crosstalk, simultaneous measurement, real time measurement, rock wool, agriculture

## Abstract

Making several simultaneous measurements with different kinds of sensors at the same location in a solution is difficult because of crosstalk between the sensors. In addition, because the conditions at different locations in plant beds differ, *in situ* measurements in agriculture need to be done in small localized areas. We have fabricated a multimodal sensor on a small Si chip in which a pH sensor was integrated with electrical conductivity (EC) and temperature sensors. An ISFET with a Si_3_N_4_ membrane was used for the pH sensor. For the EC sensor, the electrical conductivity between platinum electrodes was measured, and the temperature sensor was a p-n junction diode. These are some of the most important measurements required for controlling the conditions in plant beds. The multimodal sensor can be inserted into a plant bed for *in situ* monitoring. To confirm the absence of crosstalk between the sensors, we made simultaneous measurements of pH, EC, and temperature of a pH buffer solution in a plant bed. When the solution was diluted with hot or cold water, the real time measurements showed changes to the EC and temperature, but no change in pH. We also demonstrated that our sensor was capable of simultaneous *in situ* measurements in rock wool without being affected by crosstalk.

## 1. Introduction

Performing several simultaneous measurements with different types of sensor in conducting solutions is difficult, because of the crosstalk between sensors mediated through the solution. A sensor that applies an electrical current to the solution and another that monitors voltage potential can easily affect each other. Therefore, to make simultaneous multimodal measurements at a localized point using several kinds of sensor we need to carefully consider the operating methods used in order to avoid crosstalk.

In recently years, precise control of the growth conditions for plants has become an active area of research for ensuring food safety [1,2], increasing food production [3,4], and decreasing the labor load on agricultural workers [5]. The key point to accomplish these goals is the proper use of sensors. Numerical measurements that quantify the changes to plants and the growth environment using the sensors are required. Various kinds of sensor are used in agriculture, but these sensors are insufficient for the precision control required. Especially, *in situ* monitoring of plant beds, such as measurements of the nutrient concentration, pH, *etc.*, is difficult because particle sizes and plant roots, which are with the solution, are non-uniform [6–9]. Therefore, for *in situ* measurements in plant beds, several different types of sensor are needed to make simultaneous measurements at the same point. In addition, the size of the sensors needs to be much smaller than the sensors currently used, which are more than a centimeter in length, in order to make observations at localized points, thereby complex changes to the various conditions at different points around the roots.

**Figure 1.**
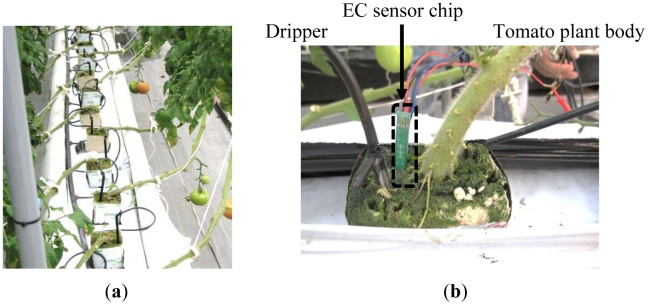
Tomato plants in a soilless rock wool medium for precision agriculture. (**a**) Tomato plants with a dripper in each piece of rock wool; (**b**) An example of *in situ* EC monitoring in rock wool using a previous EC sensor. The sensor size could be inserted without constraint.

We focused on pH, electrical conductivity (EC), and temperature sensing in soilless agriculture. These data are very important for precision agriculture [10,11]. The pH is controlled to prevent barrier growth. The measurement is important because the solubility of minerals in acidic and alkaline solutions is different and the solution concentration changes with solubility [12–15]. The EC is measured to obtain the ion concentration of all the species in the nutrient solution [16]. In precision agriculture, the composition ratio of the main ion species, *i.e.*, nitrogen ions, phosphoric acid and potassium, in the nutrient solution is known by the user. Therefore, measurement of the total ion concentration is sufficient [17]. The temperature is controlled for conservation of growing temperatures of each kind of plant [18–20], and is used to make corrections to the pH and EC. To monitor these data in soilless rock wool, as shown in Figure 1(a), it is necessary make simultaneous measurements at the same point near roots. In addition, the sensor size should be small for insertion into the medium. In other groups, EC sensor [21,22] and pH sensor [23] to monitor into medium had been studied. However, there are currently no sensors available that can perform these types of measurements.

In this study, we worked toward implementing a multimodal sensor with these three functional capabilities. This new study followed on from our previous work on a small EC sensor [24,25], which we inserted into rock wool (shown in Figure 1(b)), and an integrated EC and temperature sensor [26,27] used for monitoring the health of cows.

## 2. Design Section

In this section we discuss the planning and design of the sensors. In Section 2.1, the concept of this work is presented. In Sections 2.2 to 2.4, the sensors—pH, EC, and temperature sensors—and their fundamental measurement methods are discussed. In Section 2.5, the operation of the integrated multimodal sensor is described.

### 2.1. Concept of This Work

In our previous work, we were able to insert an EC sensor chip [24] into rock wool, as shown in Figure 1(b). In this work, *in situ* measurements in rock wool were made. The chip size in this study was 5 mm × 5 mm square which is same size as the EC sensor chip. An integrated EC and temperature sensor chip was fabricated for research in a different field [26]. This technology was used in this new study, for which multimodal sensor chips with pH, EC and temperature sensing areas were fabricated. The pH sensor on our new chip uses an ion-sensitive field effect transistor (ISFET) type sensor. This type of device is compatible with Si large-scale integration (LSI) processes. Because our temperature sensors are p-n junction diodes, these could also be fabricated with the process. The EC and pH sensors are required to make measurements at the same point in the solution. The temperatures of the solution, the air, the culture medium and so on also have to be monitored. Therefore, the placement of the sensor areas on the chip is an important design consideration. This placement is described in Section 3.

### 2.2. pH Sensor

The pH sensors fabricated using Si LSI process are mainly ISFETs [28,29] or sensors using charge transfer technology [30]. For simultaneously sensing pH and EC, an ISFET type pH sensor which can operate continuously was selected. To eliminate crosstalk it was necessary to use a band pass filter. This is discussed in Section 4. In addition, the operation is simple and miniaturization is easy.

The voltage potential *φ_ch_* of a sensing membrane in an aqueous solution obeys the Nernst equation, which is given by Equation (1) with respect to H^+^/OH^−^ions [31]:

(1) $$\varphi_{ch}=V_{\text{REF}}+\frac{RT}{F}\ln\alpha_{H}$$

where *F* is the Faraday constant, *R* is the gas constant, *T* is the absolute temperature, *V_REF_* is the potential of the reference electrode, and *α_H_* is the active ion ratio of hydrogen to hydroxide. The term *φ_ch_* is a direct current (DC) signal. This is a key point for simultaneous measurements with an EC sensor, since the EC sensor operates with an alternating current (AC) signal at 10 kHz. However, because the EC signal appears in *V_REF_* as flicker noise, simultaneous measurements are not easy to do. Nevertheless, this problem can be solved. Several kinds of membrane material have been used [32] such as Si_3_N_4_, Ta_2_O_5_, Al_2_O_3_, SiO_2_, and so on. The membrane is required to be highly non-permeable. In this study, Si_3_N_4_ was used because it is non-permeable and is compatible with Si LSI processes.

### 2.3. EC Sensor

The EC sensor uses platinum electrodes, which we have studied in previous work [24]. The sensor measures the conductivity of the solution, which gives the ion concentration of the nutrient solution in between the electrodes. Because platinum is chemically stable, long term monitoring for over 1.5 years using our EC sensor has been achieved [8]. The sensor operates at 10 kHz sine wave in order to minimize the impedance of the electric double layer which has a capacitance of about 1 μF. The EC sensor applies a voltage to the solution, and the pH sensor monitors the potential of the sensing membrane. In addition, the potential window of platinum for which no oxidation or reduction occurs is from about +0.5 to −0.5 V *vs.* SCE with pH from 0 to 14 [33]. Taking this into consideration, the EC sensor was operated with voltage amplitude of 0.25 volts and a DC offset of 0 volts.

### 2.4. Temperature Sensor

We fabricated a sensor with temperature and EC sensing areas for monitoring the health of cows in previous work [26]. The temperature sensor was a p-n junction diode which could be fabricated together with the ISFET using Si LSI technology. The forward current of a p-n junction diode is given approximately by Equation (2) [34]:

(2) $$I_{F}=I_{0}\left\{\exp\left(\frac{qV_{F}}{\text{nkT}}\right)-1\right\}$$

where *I_F_* is the forward current, *V_F_* is the forward voltage, *I_0_* is reverse saturation current, *k* is the Boltzmann constant, *T* is absolute temperature, *q* is the electronic charge, and *n* has a value between 1 and 2.

When a constant current is applied to a p-n junction temperature sensor, Equation (2) can be rearranged as follows:

(3) $$V_{F}=\frac{\text{nkT}}{q}\cdot \ln\left(\frac{I_{F}}{I_{0}}+1\right)\propto T$$

**Figure 2.**
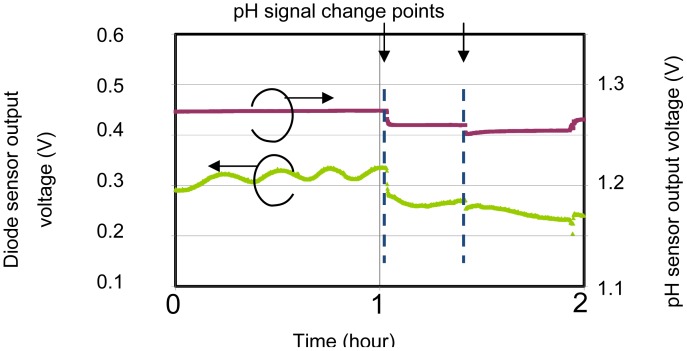
Diode signal affected by changes in the pH signal without a layer shielding the diode.

Thus, the output voltage *V_F_* varies linearly with temperature. The top passivation layers (SiOx and SiN) were used to isolate the temperature sensor from the solution. The sensor was surrounded by a p-type diffusion layer in order to isolate and shield it from noise arising from changes in the potential of the pH sensor, leakage current from the EC sensor and other external sources. Figure 2 shows that, without shielding, the sensor is affected by changes in the pH signal. When the pH signal changes, the temperature signal also changes by it. Therefore, the shielding layer is needed to stabilize the sensor potential for simultaneous measurements, because the sensitivity of the temperature sensor is only small (about 1 mV/°C).

### 2.5. Multimodal Sensor

In Sections 2.2 to 2.4 we described the characteristics of each sensor and their limitations. The operating conditions for the integrated multimodal sensor are based on these, and are shown in Table 1.

The EC sensor operates at an AC voltage of 0.25 V at 10 kHz with a zero bias offset, because the voltage should be within the range of the potential window. The pH sensor uses constant voltages for power and for the reference voltage, *V_REF_*, given in Equation (1). The reference voltage sets the pH sensor operating point by controlling the solution voltage. The pH sensor was designed as a depletion type ISFET which can be operated at zero bias, so that it would not have an impact on the offset bias of the EC sensor. In addition, for the medium culture and soil are almost ground level zero volts, the zero bias operation are important to *in situ* measurement.

In another point, DC output signal of the pH sensor is affected by the AC voltage supplied to the EC sensor. However, the AC signal affects only *V_REF_*, not *α_H_*. Therefore, this crosstalk can be removed by using a band pass filter because the EC voltage is AC and the pH signal is DC. So, the output signal of the pH sensor is filtered to remove crosstalk. The result of using the filter is shown in Section 4.

## 3. Fabrication of a Multimodal Sensor Chip and Signal Processing Circuit Module

In this section we describe the structure of the sensor chip and the signal processing circuit module. In Section 3.1, we describe the integrated multimodal sensor chip, incorporating pH, EC and temperature sensing areas. In Section 3.2, the architecture of the module is explained.

### 3.1. The Integrated Multimodal Sensor Chip

**Figure 3.**
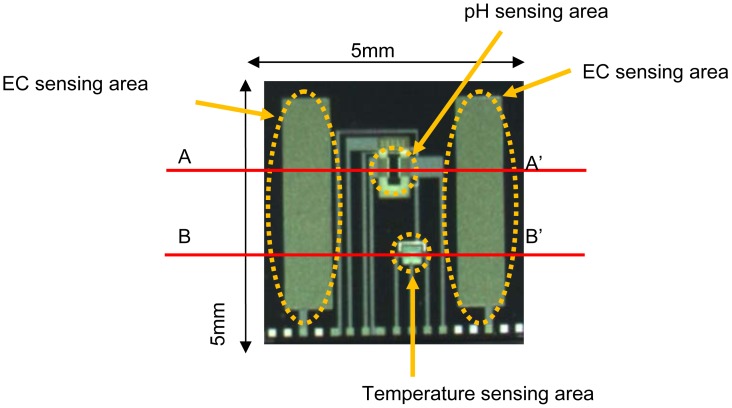
Photograph of an integrated multimodal sensor chip with pH, EC, and temperature sensing areas.

Figure 3 shows a photograph of the 5 mm × 5 mm multimodal sensor chip. The integrated chip with pH, EC and temperature sensing areas was successfully fabricated using a Si LSI process. The pH and temperature sensing areas are between the electrodes of the EC sensing area. The area is too small for medium culture. So the sensor can monitor pH, EC and temperature almost at the same point in agriculture.

**Figure 4.**
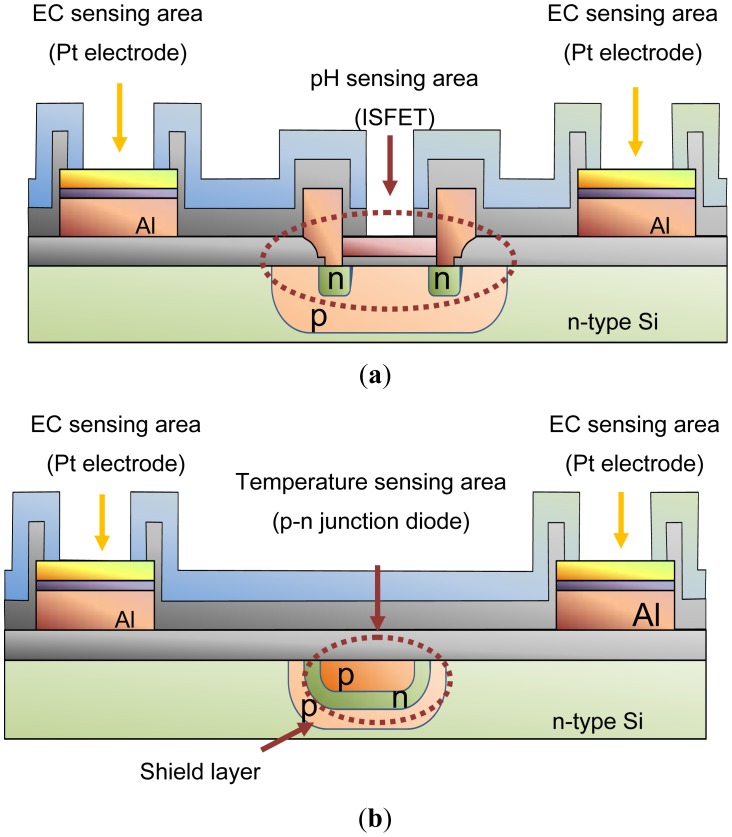
Schematic cross-sections through the multimodal sensor chip. (**a**) The pH and EC sensing areas through A-A' in Figure 3. The pH sensing area is between the Pt electrodes; (**b**) The temperature and EC sensing areas through B-B' in Figure 3. The temperature sensing area is also between the electrodes.

Figure 4 shows cross-sections of the structure at A-A′ and B-B′ in Figure 3. As seen in Figure 4(a), the pH-sensing region of the device proposed here consists of three layers: Si_3_N_4_ (ion-sensitive membrane)/SiO_2_/p-type Si substrate. The voltage potential of the Si_3_N_4_ film is proportional to the pH, and the voltage potential for values of pH between 1 and 9 can be measured [35]. So that the operating point was at zero volts, a depletion type ISFET was designed. The p-Well layer is connected to source electrode of ISFET for unaffected by substrate bias change and disturbance. Aluminum (Al) is used for electrical connections to the Pt electrodes of the EC sensing area. Silicon dioxide (SiO_2_) is used to isolate the electrodes from the Si substrate. The thickness of this layer is 1 μm or more, because the leakage current from the sensor to the Si substrate during operation has to be small. Figure 4(b) shows the structure of the temperature sensing area. This is a p-n junction diode in a p-type diffusion layer which was fabricated together with the ISFET using the Si LSI technology. Passivation layers of SiOx and SiN are used to isolate the temperature sensor from the solution. The temperature sensor is shielded by the p-type diffusion layer to isolate it and shield it from noise arising from changes in potential of the pH sensor, leakage current from the EC sensor and other external sources. The shielding layer is needed to stabilize the sensor potential for simultaneous measurements, because the sensitivity of the temperature sensor is only small (about 1 mV/°C). The shielding layer is needed connected to cathode of diode for them.

### 3.2. A Signal Processing Circuit Module

**Figure 5.**
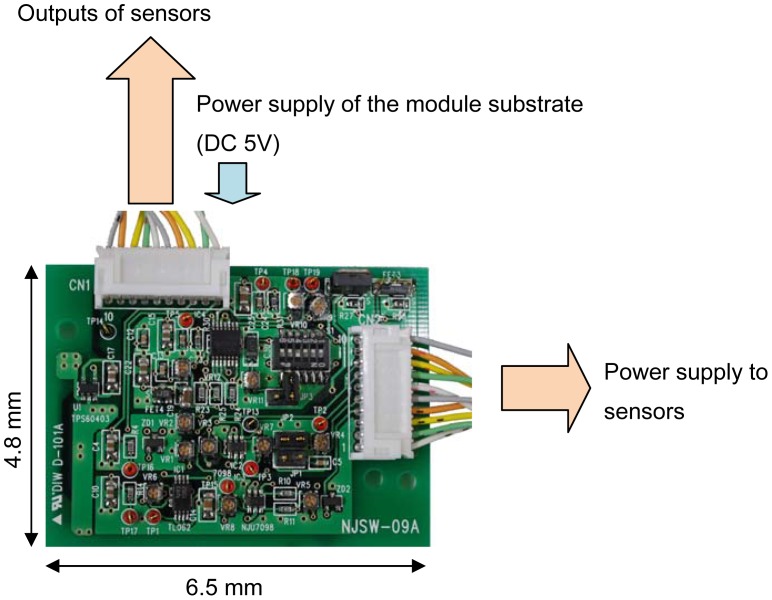
Photograph of signal processing circuit.

For simultaneous measurements, an analog signal processing circuit using discrete components was fabricated on a PCB (Figure 5). The board includes filters (high-pass filter for the EC sensor, low-pass filter for the pH sensor), a power supply (10 kHz sine wave for the EC sensor, DC voltages for the pH and temperature sensors) and amplifiers (variable gain between 5 and 50) for each sensor. Input ranges, which are between 1 and 2 volts from pH sensor, 100 and 100 k ohm of EC, and 0.1 and 1 volts of temperature, can change output voltages between 0 and 2. In addition, an AC to DC converter circuit was fabricated on the board because we used multi-channel voltages recording for the real time measurements.

## 4. Results and Discussion

In this section we show the results of measurements made with the sensor chip. In Section 4.1, the individual characteristics of each sensor are shown to confirm the measurement ranges. In Section 4.2, simultaneous measurements of a pH buffer solution were made with the multimodal sensor to demonstrate the elimination of crosstalk. In Section 4.3, we describe *in situ* monitoring in rock wool and investigate the capability of the sensor for making measurements in a culture medium.

### 4.1. Individual Characteristics of the pH, EC and Temperature Sensing Areas

**Figure 6.**
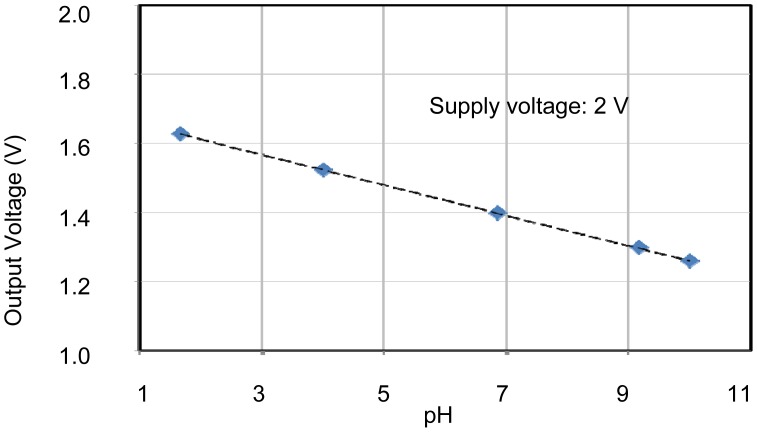
Characteristics of pH sensing area. The sensitivity of 43.8 mV/pH is sufficient for use as a pH sensor.

**Figure 7.**
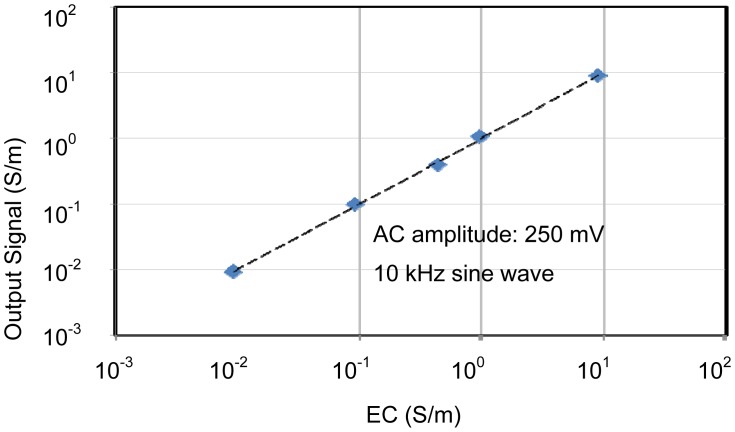
Characteristics of EC sensing area. The operating range is wide enough for use in agriculture.

**Figure 8.**
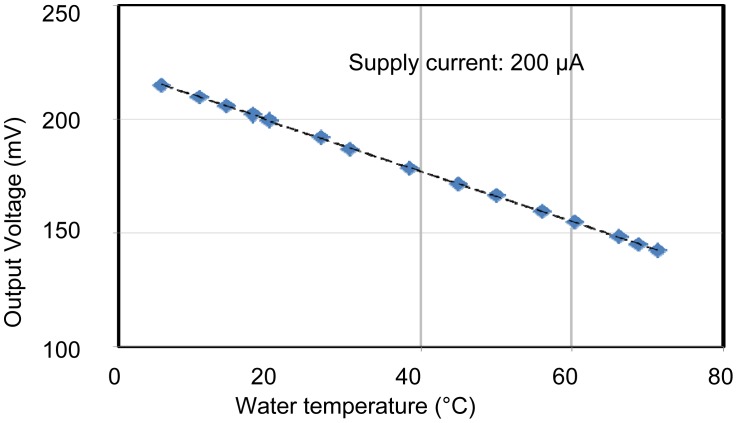
Characteristics of the temperature sensing area. It has a good sensitivity of 1.1 mV/°C and good linearity.

The individual characteristics of each sensing area were confirmed. In each case, measurements were made with the other sensors disconnected, for example, when operating the pH sensing area, no voltages were applied to the EC and temperature sensors. Therefore, the individual results shown in Figures 6 to 8 were stable and without crosstalk. Ranges, which are EC between 0.007 and 5 S/m, temperature 5 and 50 °C, and pH 2 and 9, are almost needed in agriculture. The results of sensors show that the measurement ranges and linearity are sufficient for use in agriculture.

The pH sensor was capable of measurements between about pH 2 and pH 10. This it can be used to monitor almost any crop. The EC sensor had a wide measurement range from 10 mS/m (tap water) to 10 S/m (sea water). This is sufficient for use in agriculture. It was confirmed that the temperature sensor could be used to monitor temperatures between 5 and 70 °C, which is sufficient for agricultural needs. These results confirm the successful fabrication of the multimodal sensor.

Long-term stability of their sensors had been confirmed by way of experiments. EC sensor operation for almost a year and 7 months has been achieved [8]. The temperature sensor was confirmed to measure stably for a year. The pH measurements worked for a week. The stability of the pH sensor is obviously not enough to operate in agriculture. However, a study for long-term pH experiment has been proceeding in our group. A report of our results will be presented in the future.

### 4.2. Simultaneous Measurements Using a pH Buffer Solution

**Figure 9.**
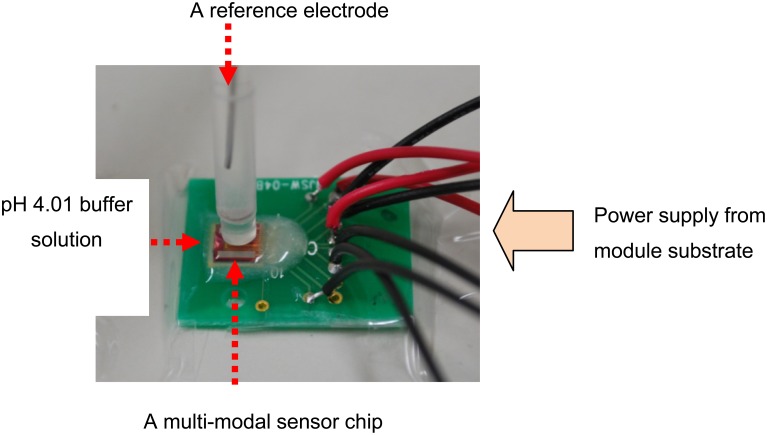
Photograph of the measurement set up for the multimodal sensor chip.

The multimodal chip and the analog processing circuit shown in Figure 5 were used to make simultaneous measurements in real time in order to check for the elimination of crosstalk between each sensor. Figure 9 shows a photograph of the measurement set up with a reference electrode and pH 4.01 buffer solution. The pH sensor electrode was used to maintain the voltage of the solution at zero. One characteristic of the buffer solution is that there is no change in pH when the solution is diluted. In this experiment, it was shown that the pH did not change, but the EC and temperature measurements changed when the buffer solution was diluted with tap water.

**Figure 10.**
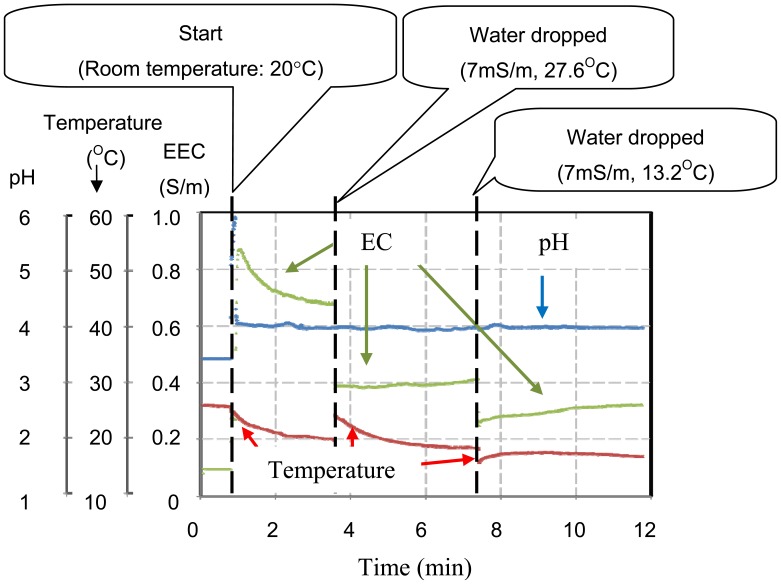
Results of measurements using the multimode sensor.

Figure 10 shows the results of measurements of pH, EC, and temperature. Initially, the chip was dried at 26 °C, then 100 μL of pH 4.01 buffer solution was introduced between the multi-modal sensor and the reference electrode at 20 °C. After 3.6 min, the measurements recorded by the chip were pH 4.0, EC 0.82 S/m and temperature 19.8 °C. At 3.6 min, hot tap water (volume: 50 μL, pH: 7, EC: 7 mS/m, temperature: 27.6 °C) was added to the solution. Following this, the EC measurement fell to 0.46 S/m and the temperature rose to 24.4 °C. However, the pH remained unchanged. After 7.4 min, cold tap water (volume: 30 μL, EC: 7 S/m, temperature: 13.2 °C) was added. The EC measurement changed again to 0.29 S/m, and the temperature fell to 15.8 °C. As before, the pH remained the same. These results show that there was no crosstalk between the sensors and correct sensing operation was achieved. When the pH 4.0 solution was diluted by tap water, the EC and the temperature changed, but the pH remained unchanged. These results are characteristic of the buffer solution. Successful real time and simultaneous measurements were made using the multi-modal chip.

### 4.3. Simultaneous Measurements in Rock Wool

**Figure 11.**
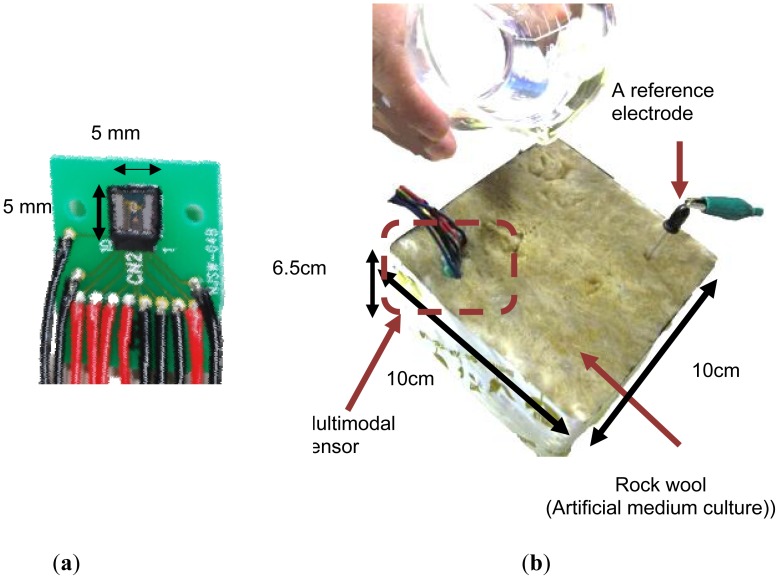
Photograph of the sensor chip and the experiment. (**a**) The 5 mm × 5 mm sensor chip bonded and electrically-wired to a PCB substrate. The connections between the chip and the PCB, and between the PCB and the lead wires are shielded by epoxy resin and adhesive bonds; (**b**) The sensor and the reference electrode are inserted in the rock wool. The rock wool includes a nutrient solution diluted by 1%.

The multimodal sensor was inserted into rock wool to conduct *in situ* simultaneous real time monitoring of three important quantities used in precision agriculture. Figure 11 shows a photograph of the sensor chip and the experiment. A nutrient solution used in agriculture was diluted by 1%. Plenty of the solution was added to the rock wool initially.

The position of the reference electrode was checked for its impact on the pH measurement. When the distance between the sensor and the electrode was changed from 1 cm to 12 cm, the stability of the pH measurement was about pH 0.02 which is sufficient for agricultural purposes. Therefore, the measurement was not limited by the position of the electrode. Additionally, for removal reference electrode, reference field effect transistor (REFET) [36] and *etc.* had been studied in other groups. The reference electrode will be no need to operate in pH measurements.

**Figure 12.**
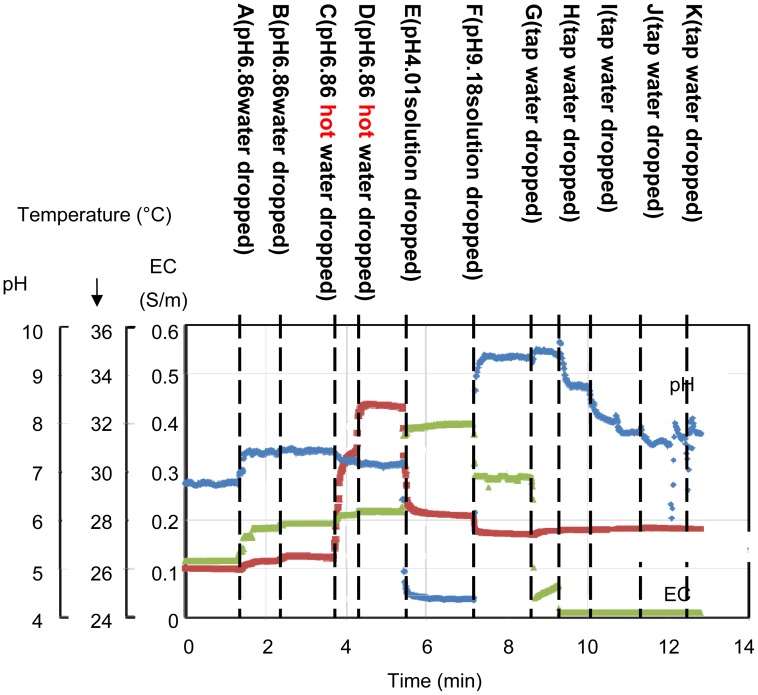
Results of experimental *in situ* measurements. Various 25 mL solutions were dropped onto the rock wool.

Figure 12 shows the experimental results of the *in situ* measurements. Table 2 shows the data for the results shown in Figure 12. Various 25 mL solutions were dropped onto the rock wool. First, a pH 6.86 buffer solution diluted by 22%, in which pH buffering effect of the solution becomes weak, was added at points A and B. The pH changes from pH 6.8 to 7.39 at point A and remains at pH 7.39 at point B. When a warm solution was added at point C, the temperature changed from 26.5 to 30.7 °C. At point E, a pH 4.01 buffer solution without dilution was added. The pH sensor output changed to 4.38, and the temperature output changed to 28.2 °C after adding the colder solution. The EC characteristics of the solution rise from 0.115 S/m at A to 0.396 S/m at E. At F, a pH 9.18 buffer solution without dilution was added. The pH output changed to 9.39, and the EC output changed from 0.396 to 0.288 S/m, which is smaller than the EC at pH 4.01. From points G to K, tap water was added. As the solution in the rock wool is gradually diluted, the EC output decreases and the pH output moves towards a neutral pH value. The reactions of the sensors to the added solutions are shown in Table 2. These results show significant changes in sensor output in response to the added solutions. In actual fact, the sensor outputs were not completely in accord with the characteristics of the mixed solutions included in the rock wool. However, the results show that the sensor is capable of continual surveillance of a culture medium in real time, and *in situ* monitoring is important to gain an understanding of the optimum conditions. In this experiment, the sensor was inserted near the surface of the rock wool. Therefore, quick responses to the added solutions were obtained. If the sensor was inserted at the base in an actual cultivation environment, the information gained would be of unused nutrients.

**Figure 13.**
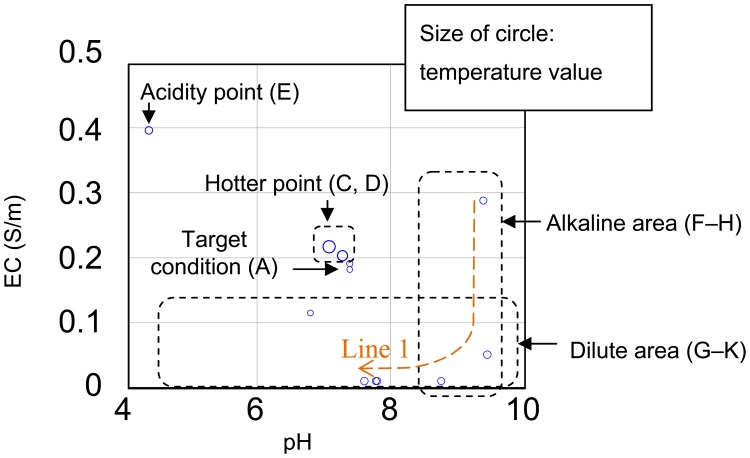
Comparing results of pH, EC and temperature in sensor outputs of Table 2 is shown. The size of circle was varied by temperature value, for example, smallest circle is 26.0 °C, and largest circle is 32.7 °C.

Figure 13 is shows to compare results in sensor outputs of Table 2. If the point A of Table 2 is ideal, other points which are hotter point, acidity point, alkaline area and dilute area need adjustment of conditions. When E of alkaline area was diluted for toward neutral pH, the pH achieved it in exchange for decrement of EC in line 1 which were E though K. Their speculations showed of efficacy of multimodal sensor. Additionally, agricultural workers will be able to visualize of condition in medium culture by using the sensor.

## 5. Conclusions

An integrated multimode sensor for simultaneous measurements of the pH, EC and temperature of a conducting solution was proposed. Crosstalk through the solution and the chip substrate makes it difficult to make simultaneous independent localized measurements for different operating methods of the sensors. However, *in situ* measurements in non-uniform media are needed in order to do a proper analysis. The sensor structure and operating conditions were designed to solve this problem. We successfully fabricated multimodal sensors using a Si LSI process. We also made a signal processing circuit using discrete components. Simultaneous measurements of pH, EC and temperature in a pH 4.01 buffer solution and in rock wool were demonstrated. The experimental results showed that the sensor could be used to simultaneously measure pH, EC and temperature in the same area without limitation. A usable sensor for precision control in agriculture was achieved.

## Figures and Tables

**Table 1.** Operating conditions for pH, EC and temperature sensing areas.

**Characteristics of Signal**		**EC Sensor (Pt electrodes)**	**pH sensor (Depletion Type ISFET)**	**Temperature Sensor(p-n Junction Diode)**
Electrical power supply to sensor	DC phase	0 V	Constant voltage for source (Extra supply: Reference voltage should be 0 V)	Constant current
	AC phase	10 kHz sine wave (Amplitude: 0.25 V)	-	-

Output signal from sensor	DC phase	-	Change of voltage	Change of voltage
	AC phase	Change of amplitude voltage	-	-

**Table 2.** The data in Figure 12 is shown in the table in order to compare the reaction of the sensors to the added water. The initial nutrient solution was diluted to a density of 1%, A, B, C and D were pH6.86 buffer solutions diluted to a density 22%, E was a pH 4.01 buffer solution without dilution, F was a pH 9.18 buffer solution without dilution, and G to K were tap water.

**Point**	**Characteristics of Solutions**			**Sensor Outputs**		

	**EC (S/m)**	**Temperature (°C)**	**pH**	**EC (S/m)**	**Temperature (°C)**	**pH**
Initial	0.120	26.6	6.10	0.115	26.0	6.8
A	0.190	27.1	7.05	0.182	26.3	7.39
B	Same as above	Same as above	Same as above	0.191	26.5	7.39
C	0.190	35.6	7.05	0.203	30.7	7.28
D	Same as above	Same as above	Same as above	0.217	32.7	7.08
E	0.502	25.5	4.01	0.379	28.2	4.38
F	0.186	25.7	9.18	0.288	27.4	9.39
G	0.00808	27.8	8.03	0.0509	27.8	9.45
H	Same as above	Same as above	Same as above	0.0102	27.6	8.76
I	Same as above	Same as above	Same as above	0.0102	27.6	7.78
J	Same as above	Same as above	Same as above	0.0103	27.7	7.61
K	Same as above	Same as above	Same as above	0.0102	27.6	7.80
